# Structural Modifications of siRNA Improve Its Performance In Vivo

**DOI:** 10.3390/ijms24020956

**Published:** 2023-01-04

**Authors:** Ivan V. Chernikov, Ulyana A. Ponomareva, Elena L. Chernolovskaya

**Affiliations:** Institute of Chemical Biology and Fundamental Medicine, Siberian Branch of the Russian Academy of Sciences, Acad. Lavrentiev Ave., 8, 630090 Novosibirsk, Russia

**Keywords:** siRNA, DsiRNA, chemical modifications, branched siRNA, multimeric siRNA, structure-function relationship, suplamolecular complexes

## Abstract

The use of small interfering RNA (siRNA) in the clinic gives a wide range of possibilities for the treatment of previously incurable diseases. However, the main limitation for biomedical applications is their delivery to target cells and organs. Currently, delivery of siRNA to liver cells is a solved problem due to the bioconjugation of siRNA with N-acetylgalactosamine; other organs remain challenging for siRNA delivery to them. Despite the important role of the ligand in the composition of the bioconjugate, the structure and molecular weight of siRNA also play an important role in the delivery of siRNA. The basic principle is that siRNAs with smaller molecular weights are more efficient at entering cells, whereas siRNAs with larger molecular weights have advantages at the organism level. Here we review the relationships between siRNA structure and its biodistribution and activity to find new strategies for improving siRNA performance.

## 1. Introduction

RNA interference (RNAi) is an evolutionarily conservative biological process of suppression of gene expression, implemented at the post-transcriptional level [1]. Therefore, RNAi is a very convenient method for suppressing gene expression in experimental biology and clinical practice; five drugs based on small interfering RNAs have been approved by the FDA and are commercially available [2,3]. However, a huge number of potential siRNA-based drugs have failed clinical trials because many factors limit the use of siRNA in biomedicine, such as the sensitivity of siRNA to the action of ribonucleases, filtration by the kidneys, uptake by immune cells, off-target effects, low efficiency of penetration through a hydrophobic cell membrane, and release of siRNA from endosomes.

The use of siRNA bioconjugates is the most promising method for siRNA delivery, as it is characterized by low toxicity and high efficiency in vivo. Currently, most drugs from the pipeline of leading pharmaceutical companies (Alnylam Pharmaceuticals, Silence Therapeutic, Arrowhead Pharmaceuticals, Dicerna Pharmaceuticals) are siRNA and N-acetylgalactosamine conjugates and are designed to deliver siRNA to liver cells, so we can assume that siRNA delivery to this organ is a solved problem (reviewed in [4,5]) since the addition of N-acetylgalactosamine to siRNA contributes to its significant accumulation in hepatocytes and provides a long-term effect. Therefore, the search for new molecules to conjugate with siRNA to deliver siRNA to other organs is one of the most urgent tasks (reviewed in [6,7]). However, the accumulation in the target organs and the overall biodistribution of bioconjugates of nucleic acids in the body and cells depend not only on the delivery molecule but also on the molecular weight and structure of siRNA [8]. Nucleic acids with a molecular weight smaller than the classic 21 bp duplex, such as ASO, are better accumulated within cells, while longer >21 nt dsRNAs tend to have benefits at the organism level. In this review, we will analyze available literature data on the relationship of the performance of siRNA bioconjugates with their structure.

## 2. Mechanism of RNA Interference

**Figure 1:**
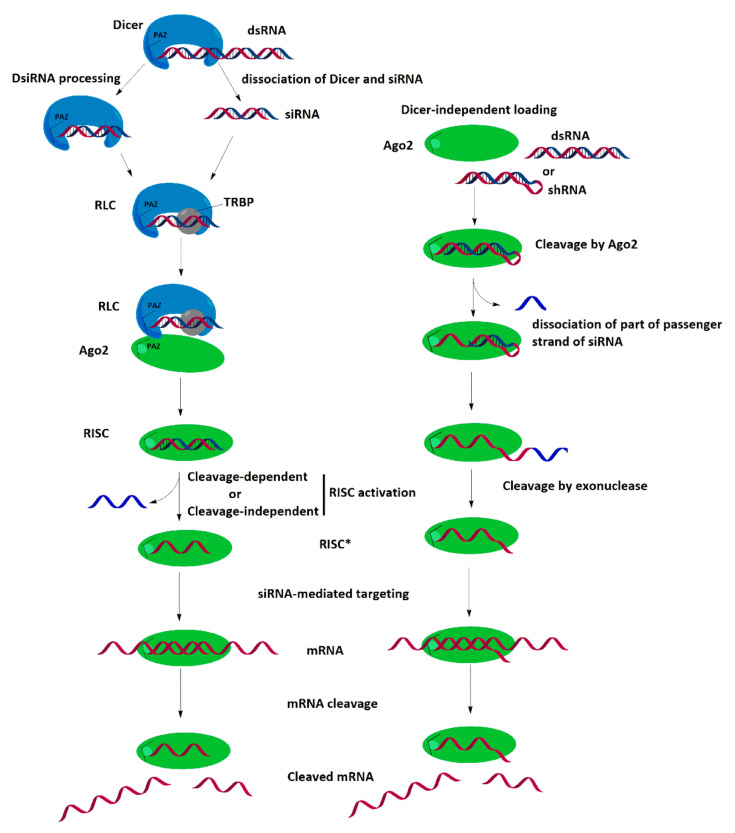
Dicer-dependent and Dicer-independent mechanisms of RNA interference.

The requirements for the structure of siRNA are determined by the mechanism of RNAi and the structure of the proteins involved in it. The mechanism of RNA interference can be divided into two stages: the initiation phase, cleavage of long dsRNA into short fragments (siRNA), and the effector phase, the formation of the activated RISC* complex. At the first stage, the dsRNA that entered the cytoplasm is cut by the Dicer enzyme into short fragments 21–23 bp long with two nucleotides protruding at the 3′-end [9]. After that, Dicer and siRNA dissociate; however, it is assumed that under certain conditions, Dicer can remain associated with siRNA, facilitating its further processing (Figure 1) [10].

At the second stage of RNAi, a dimer consisting of Dicer and TRBP interacts with siRNA and forms an intermediate RLC complex, with TRBP binding to the thermodynamically more stable end of the siRNA duplex, and Dicer to the less stable one [11,12]. At the next stage, the Ago2 protein binds to the siRNA, displacing the protein dimer from the RLC, while the MID and PIWI domains of Ago2 bind the 5’-end of the “guide” strand of the siRNA, where TRBP is located [13], while the PAZ domain is attached to the 3’ end of this strand, where Dicer is located [14], forming the RISC complex. This is followed by local unwinding of the duplex and cutting of the “passenger” strand between the ninth and tenth nucleotides from the 5′-phosphate of the “guiding” strand by the PIWI domain of Ago2, which has an RNase H activity. After the “passenger” strand dissociates from the complex, an activated RISC* complex is formed, which includes Ago2 and the “guiding” siRNA strand.

Thus, for a 21 bp siRNA, the thermodynamic asymmetry of the duplex ends is one of the key factors determining the emplacement of the siRNA in the RLC complex and the choice of the “guide” strand in RISC* [15]. Another strand selection mechanism can be implemented for dsRNAs longer than 21 bp, which are Dicer’s substrate; it is assumed that since Dicer may not dissociate after cleavage, but remain associated with the protruding 3’-end of siRNA, then it will spatially orientate the RLC formation and, accordingly, determine the choice of the “guide” circuit that will be included in RISC* (Figure 1). Finding the optimal structure for Dicer Substrate Small Interfering RNA (DsiRNA) recognition to incorporate the antisense strand into RISC* is a developing field of research [10].

It should be noted that the incorporation of siRNAs into RISC* can be carried out without Dicer’s participation. Here, dsRNA forms a complex with Ago2 by direct loading: during this process, the 5’-phosphate-binding pocket formed by the MID and PIWI domains of Ago selectively binds the 5’-terminal phosphate of the nucleotide located at the less stable end of the siRNA duplex via a Dicer-independent pathway [16] (reviewed in [17,18]).

## 3. Chemical Modifications

Chemical modifications are essential for the use of siRNA in bioconjugates in vivo since siRNA is sensitive to ribonucleases and, unlike liposomes and nanoparticles, is not protected in the conjugate; this topic is widely discussed in numerous reviews [6,19,20]. Chemical modifications of siRNA are an actively developing field; in short, there are modifications of ribose (LNA, UNA, GNA, CeNA, HNA, 2’O-Me, 2’O-MOE, 2’F and 4′-thioribonucleosides [21,22,23,24,25,26,27]), phosphate (Tert-butyl-S-acyl-2-thioethyl, boranophosphate, 5′(E)-vinylphosphonate, phosphorothioate [PS] [28,29,30]) and base modifications (pseudouridine, 2′ thiouridine [31]). Ribose modifications affecting the 2’OH group are the most effective in blocking endoribonucleases, which make the main contribution to siRNA cleavage, whereas phosphate modifications block exonucleases better. The cleavage mechanism by endonucleases involves the 2’OH group of the ribose, so replacement of all this group with a 2’F fluorine or 2’O-Me group is necessary throughout the duplex. It was shown that the introduction of alternating 2’F and 2’O-Me into both strands of siRNA does not block its interaction with RISC components and increases the duration of gene silencing. The bulkier 2’O-Me modification effectively protects siRNA from the action of nucleases; however, this modification more significantly reduces the activity of RNAi, especially when applied at certain positions of the duplex [32,33]. Therefore, a balance between the number of 2’O-Me and 2’F modifications is necessary to ensure the effectiveness of siRNA action. To achieve this goal, attempts were made to increase the proportion of 2’O-Me modifications in siRNA by reducing the proportion of 2’F in the alternating pattern of modifications [34,35]. It has been shown that a decrease in the proportion of 2’F to 18% increases the efficiency and duration of action of such siRNAs in GalNAc conjugates. The modification pattern [34] was chosen as a statistical consensus on 1890 siRNAs and does not usually block the RNAi process for most siRNAs; further optimization of the 2’F/2’O-Me composition for each siRNA sequence can increase its activity [4,36,37]. Detailed optimization of 2’F/2’O-Me composition was carried out for asymmetric anti-*FLT1* siRNA [38], and it was shown that the introduction of 2’O-Me at all nucleotides except two 2’F (6%) does not impair the silencing activity of siRNA in vitro and increases the accumulation in the liver, kidney, and placenta of the lipophilic conjugate of this siRNA compared to siRNA containing 50% 2’F. The duration of the biological effect of such an optimized siRNA was not specified by the authors.

For the optimization of 2’F/2’O-Me modifications, it should be considered that some positions of 2’F are probably more significant—for example, it was shown that the 14th 2’O-Me instead of 2’F in the antisense strand of fully modified siRNA is not tolerated by RNAi machinery [39]. Several 2′Fs in the center of sense strand (9–11 nt) are preferred, probably due to Ago2 cleavage of the passenger strand at this location and more efficient release of the passenger strand from RISC (Table 1) [34].

For increasing the 2’O-Me/2’F relation in the case of long fully modified dsRNAs, which are Dicer’s substrate, it should be taken into account that the slicing activity of Dicer is blocked by 2’O-Me [40], so 2’F modifications should be placed in the sites of RNA cleavage by Dicer.

Further optimization of siRNA chemical modifications such as GNA, stereoselectivity of modifications, and Alnylam’s IKARIA platform decrease therapeutic dose and improve safety profile of siRNA and, thereby, gives us more opportunities for widespread use of siRNA in the clinic.

**Table 1:** Structural modifications of ss-siRNA.

#	Structure of siRNA—Sense Strand 5’-3’ (S) Above, Antisense Strand 3’-5’ (AS); Below, Pattern of Chemical Modifications ^1,2^	In Vitro			In Vivo			Ref.
		Cells/ Target	Delivery ^3^	Efficiency ^4^ ↑-Increase ↓-Decline	Model/ Target	Dose, Mode of Adm ^5^	Efficiency ↑-Increase ↓-Decline	
1	ss-siRNA, n/m	HaCaT/*F3*	Lf2000	87%, mRNA	-	-	-	[41]
	siRNA, n/m			90%(↑3%), mRNA	-	-	-	
2	ss-siRNA, (boranophosphate) 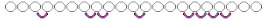	HeLa/*EGFP*	Oligofectami-ne, 25 nM	100%, protein	-	-	-	[28]
	siRNA, n/m			70%(↓30%), protein	-	-	-	
3	ss-siRNA 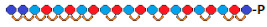	HeLa/ *PTEN*	Lf2000	IC50 2 nM, mRNA	-	-	-	[42]
	siRNA 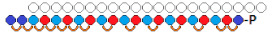			IC50 0.2 nM(↑×10), mRNA	-	-	-	
	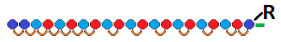 **R**: 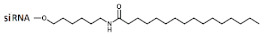	Prim. hepatocytes/*ApoC III*	Electroporation	IC50 2 mcM, mRNA	Transge-nic mice, liver/ *ApoC III*	14 mg/kg, s.c.	65%, IC50 ~10 mg/kg, mRNA	
4	ss-siRNA 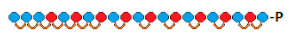	MCF-7/*PR*	RNAiMAX, 50 nM	~85%, mRNA	-	-	-	[43]
		HEK 293/*SIN3A*		5%, mRNA				
	siRNA 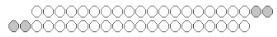	MCF-7/*PR*		~90%(↑5%), mRNA	-	-	-	
		HEK 293/ *SIN3A*		~60%(↑55%), mRNA				
5	ss-siRNA 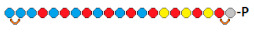	Hepa 1–6/*CTNNB1*	RNAiMAX	IC50 0.07 nM	Mice, liver/ *CTNNB1*	0.05 mg/kg i.v. with LNP	50%, mRNA	[44]
	siRNA 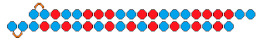			IC50 0.12 nM (↓×1.7)			65%(↑15%), mRNA	

^1^ Chemical modifications are indicated: 2’F (red), 2’O-Me (blue), 2’O-MOE (dark blue), dNMP (grey), PS (orange), 5’-(E)-VP (green), inverted thymidine (black), boranophosphate modification (purple) and LNA (yellow). n/m—non-modified. ^2^ “R”—linker with ligand. ^3^ Lf2000—transfection agent Lipofectamine 2000. ^4^ A decrease (↓) or increase (↑) in the effectiveness of a particular modification to silencing activity of siRNA. ^5^ Route of administration: i.v.—intravenously, s.c.—subcutaneously.

## 4. Single-Stranded siRNAs

A factor limiting the effective delivery of siRNAs to cells in vivo is the low ability of siRNAs to leave endosomes; therefore, to overcome this limitation, it was proposed to use single-stranded ss-siRNAs (single stranded siRNAs, ss-siRNAs). Since these molecules have a lower molecular weight, they bind efficiently to serum proteins and thus better penetrate the target cell. Such ss-siRNAs are inducers of RNAi and make it possible to avoid undesirable side effects from the side of the “passenger” strand in the case of siRNA duplexes. However, despite these advantages, unmodified ss-siRNAs have lower gene silencing activity compared to canonical double-stranded siRNAs (Table 1, #1) and are unstable in serum (cleavage time of about 5 s); therefore, related studies were aimed at creating chemically modified ss-siRNAs [41].

The first attempt to use modified ss-siRNAs was made in the work [28] (Table 1, #2), where it was shown that ss-siRNAs containing the boranophosphate modification are 30% more effective in silencing the target gene compared to unmodified siRNA duplexes; however, the stability of such ss-siRNAs remains low, requiring additional modifications. Further studies led to the creation of a pattern of chemical modifications of ss-siRNA, including 14 PS modifications and alternating 2’F and 2’O-Me nucleotide analogs [32,33], which contributes to an increase in the stability of ss-siRNA in vitro up to 8 h or more. Optimization of ss-siRNA design by introducing chemical modifications contributes not only to stability in biological fluids, but also increases the duration of ss-siRNA action. It was shown that the half-life of ss-siRNA from mouse tissue is about 10 days, and the duration of the inhibitory effect of ss-siRNA reaches 7 days [45]. The improvement of the pharmacokinetic properties of siRNA was achieved by introducing PS modifications that increase the efficiency of binding of oligonucleotides to serum proteins, which prevents the rapid elimination of ss-siRNA from the body by filtration by the kidneys [46].

A comparison of the interfering properties of a fully modified ss-siRNA and its siRNA duplex with an unmodified sense strand in vitro shows that ss-siRNA is an order of magnitude less active than the siRNA duplex when delivered to cells using a transfection agent (Table 1, #3) [42]. Chemically modified ss-siRNAs were also significantly less active than unmodified siRNA duplexes. It was shown that the modified ss-siRNA suppresses the expression of the SIN3A gene by 5%, while the unmodified siRNA duplex—by 60% when delivered to HEK293 cells using a transfection agent (Table 1, # 4) [43]. However, in in vivo experiments in mice, effective suppression of the target gene occurred in the liver by 65% (*ApoC* gene) after subcutaneous administration of ss-siRNA (Table 1, #3) [42] and in the brain by 80% (*Htt* gene) after direct intraventricular injection of ss-siRNA [47]. In another work, the biological activity of fully modified ss-siRNA and siRNA was compared in vitro and in vivo (Table 1, #5) [44]. It was shown that ss-siRNAs have a 1.7-fold lower IC50 value compared to siRNAs when delivered to Hepa1–6 cells using a transfection agent. However, after intravenous injection of 0.05 mg/kg ss-siRNA in mice in combination with lipid nanoparticles, a 50% decrease in the level of CTNNB1 gene mRNA in the liver is observed, while the biological activity of the siRNA duplex is 65%. Despite the fact that chemically modified ss-siRNAs are more effective than their unmodified counterparts, the doses required to effectively suppress gene expression remain relatively high.

## 5. Segmented siRNAs (sisiRNAs)

Another strategy to increase the specificity of siRNA is to introduce a single-strand break in the sense strand of siRNA (small internally segmented interfering RNA, sisiRNA) [21]. Such sense strand inactivation in the sisiRNA structure prevents off-target effects caused by the sense strand if it is incorrectly chosen as a “guide” and loaded into RISC*. However, the low thermal stability of sisiRNA compared to siRNA duplexes of the same sequence is their significant disadvantage; therefore, to solve this problem, it was proposed to introduce LNA modifications into sisiRNA, which would stabilize it [22]. A comparison of the biological activities of LNA-modified sisiRNA and siRNA when delivered to cells using a transfection agent showed that sisiRNA suppresses the expression of the target gene by 12% less effectively than siRNA. However, in vivo experiments on the MiaPaca-2 xenograft tumor model in mice showed that LNA-modified sisiRNA and siRNA have a similar distribution profile in tissues and accumulate predominantly in the kidneys; in addition, they exhibit similar biological activity and suppress the expression of the EGFP gene by 50% (Table 2, #6) [22].

In another study [48], dimeric sisiRNAs linked by a disulfide bond and delivered to cells by cationic polymers were studied. It is assumed that the presence of a nickname in the sense strand facilitates the formation of complexes with cationic polymers due to its structural flexibility. It was shown that after transfection of MDA-MB-435 cells expressing GFP, complexes of dimeric siRNA and sisiRNA suppress the expression of the target gene by 35% and 45%, respectively, while monomeric siRNA did not show biological activity. It is unlikely that this fact can be unequivocally assessed as evidence of the superiority of non-canonical structures; rather, it indicates an unsuccessful choice of a sequence as a non-canonical siRNA, due to which an incorrect assembly of the RISC complex occurs.

Probably, the differences between sisiRNA and siRNA are due to an increase in the specificity of action. However, since the structure of the sisiRNA duplex does not contribute to a significant increase in biological activity in vitro and in vivo, further optimization of the design of the molecule is required in order to consider it for use in the clinic.

**Table 2:** Structural modifications of sisiRNA.

#	Structure of siRNA—Sense Strand 5’-3’ (S); Above, Antisense Strand 3’-5’ (AS); Below, Pattern of Chemical Modifications ^1^	In Vitro			In Vivo			Ref.
		Cells/ Target	Delivery ^2^	Efficiency ^3^ ↑-Increase ↓-Decline	Model/ Target	Dose, Mode of Adm ^4^	Efficiency ↑-Increase ↓-Decline	
6	siRNA 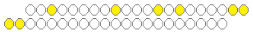	MiaPaca-2/*EGFP*	Lf2000 5 nM	~62%, mRNA	Xeno-graft MiaPaca−2 in mice/ *EGFP*	0.25 mg/kg, s.c. within 1 week, osmotic pump	50%, mRNA	[22]
	sisiRNA ss nick 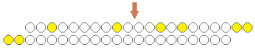			~50% (↓12%), mRNA			50%, mRNA	

^1^ Chemical modifications are indicated: LNA (yellow). ^2^ Lf2000—transfection agent Lipofectamine 2000. ^3^ A decrease (↓) or increase (↑) in the effectiveness of a particular modification to silencing activity of siRNA. ^4^ Route of administration: s.c.—subcutaneously.

## 6. Circular Small Interfering RNAs (csiRNA)

Circular RNAs (circRNA) are endogenous regulatory single-stranded, covalently closed RNA molecules that have recently attracted the attention of researchers studying their involvement in disease development as markers and potential molecular targets [49,50,51]. Recently, analogues of these natural RNAs have been proposed as synthetic inducers of RNAi—circular small interfering RNAs (csiRNAs). Natural circular RNA molecules are formed enzymatically as a result of splicing; synthetic analogues of siRNAs are usually obtained as a result of chemical crosslinking using various approaches. A number of approaches have used reversible cross-links that could break down under intracellular conditions and restore the original siRNA duplex.

Thus, in [52] the so-called caged circular RNA was proposed, sense, antisense or both strands of which were cyclized with a photosensitive crosslink using a carboxylic acid attached through a photocleavable linker at the 5’ end. They showed that such siRNAs have increased stability in the presence of serum, despite the fact that additional chemical modifications to increase nuclease resistance were not used, and are able to suppress the expression of the target gene in vitro and in vivo. Cyclization of the sense strand did not interfere with the biological activity of the duplex, while duplexes with a cyclized antisense strand were active only after photocleavage. Thus, it was shown that duplexes with a circular sense strand do not require cleavage and can be used without introducing labile bonds into their composition [53].

An alternative approach was proposed in [54], where circular duplexes were used, obtained either by enzymatic cyclization of strands containing a photo-cleavable linker in the middle or by formation of a reducible disulfide bond. It was found that changing the structure of the duplex reduces unwanted immunostimulation compared to a linear duplex of the same sequence and almost does not reduce the interfering activity.

Another interesting variant of circular macromolecular structures composed of two or three siRNA motifs was proposed in [55], where circular molecules were obtained using a template-mediated ligation method. Circular RNAs contained loops in their composition at the sites of their desired cleavage to obtain siRNAs; such cleavage, according to the authors, will occur in the cell under the action of ribonucleases. It was shown that such structures are indeed capable of suppressing the expression of the target gene by the mechanism of RNAi in cell culture.

The most advanced technology for obtaining chemically modified therapeutic siRNA conjugates was applied to circular RNAs in [56]. Fully modified duplexes were constructed in this work, containing a circular sense strand with an attached GalNAc residue and a linear antisense strand with a non-cleavable (E)-vinylphosphonate. Cyclization was carried out using copper-dependent click chemistry. Cyclization has been shown to impact potency of RNAi-mediated silencing in vitro; however, in vivo this effect was almost offset by increased metabolic stability, and the use of (E)-vinylphosphonate almost completely restored the activity. The reduction in off-target effects caused by the action of the sense strand also argues in favor of considering such a structure for the development of potential therapeutic drugs; however, so far there is not enough data to prove the safety of using such structures.

## 7. Dicer Substrates

The use of Dicer substrates (DsiRNA) as inducers of RNAi is based on the ability of Dicer to remain associated with the protruding 3’-end of the duplex after processing and facilitate the inclusion of a “guide” strand into RISC*, which makes it possible to reduce the effective concentration of siRNA required to suppress the target gene. Thus, in one of the first works devoted to non-canonical RNAi inducers, it was shown that 27–30 bp dsRNAs, which are Dicer’s substrates, have greater interfering activity compared to the 21-bp canonical siRNA. A comparison of the effectiveness of target gene suppression using DsiRNA and siRNA showed that 25–30-bp duplexes are about 100 times more active than 21-bp siRNAs (Table 3, #7) [57].

To study the effect of DsiRNA structure on recognition by Dicer, three types of DsiRNA conjugated with palmitic acid with the lengths of the sense strand 21, 23, and 25 nucleotides and the antisense strand of 23, 25, and 27 nucleotides, respectively (21/23 DsiRNA, 23/25 DsiRNA, and 25/27 DsiRNA) were synthesized [58]. It was shown that the 21/23 DsiRNA conjugate is not processed by Dicer but, nevertheless, suppresses the expression of the target gene with high efficiency (93%) (Table 3, #8). An increase in the length of the duplex by two nucleotides leads to the recognition of the 23/25 DsiRNA conjugate by Dicer; however, after cleavage, in addition to 21 bp siRNA, a mixture of products of different lengths is formed. A further increase in length to 25 nucleotides in the sense strand and 27 nucleotides in the antisense strand provides high specificity for recognition of the 25/27 DsiRNA conjugate by Dicer to form a 21 bp siRNA. Nevertheless, it was shown that, despite the differences in processing, all the conjugates studied in the work have high biological activity and suppress the expression of the target gene by 91–93%, which slightly exceeded the activity of the conjugate of the canonical 21 bp siRNA (87%).

The high biological activity of 25/27 DsiRNAs in vivo has been shown in a number of studies. Thus, the decrease in the level of mRNA of the CCR2 gene by 80% upon delivery of 25/27 DsiRNA to HEK293 cells using a transfection agent and by 71% after two intrathecal injections of 25/27 DsiRNA in rats together with CPP (transductin) (Table 3, #9) was shown in [59]. In [43,44] the interfering properties of 25/27 DsiRNA bioconjugates were studied in vivo and their high biological activity was shown. For example, the effectiveness of the DsiRNA conjugate with the aptamer was 90% after intravenous administration to mice infected with the human immunodeficiency virus (Table 3, #10) [60]. The conjugate of DsiRNA and α -tocopherol has been shown to reduce the level of mRNA of the ApoB gene in the mouse liver by 80% after 2 days (Table 3, #11) [61].

Despite the fact that Dicer has specificity for a certain duplex structure, in particular the presence of a protruding 3’ end of the DsiRNA antisense strand, it can also process DsiRNAs with blunt ends. It was shown that 27 bp long DsiRNA reduces the expression of the reporter target gene by 35% more effectively than canonical siRNA when delivered to cells with the help of a transfection agent (Table 3, #14) [62]. A comparison of the effectiveness of the conjugates of 27 bp DsiRNA and siRNA with palmitic acid in vitro and in vivo showed that both conjugates have high biological activity when delivered to cells with a transfection agent (the efficiency was 75% and 77% for the conjugates of siRNA and DsiRNA, respectively) [63]. In vivo experiments also showed a high efficiency of these conjugates: 62% and 70% gene silencing by siRNA and DsiRNA conjugates, respectively (Table 3, #15).

To assess Dicer’s specificity for DsiRNA with a hairpin, [10] constructed DsiRNAs containing a four-nucleotide looped hairpin (tetra-looped DsiRNA) at the end of the duplex (Table 3, #12). This structure was chosen on the basis of data showing that RNA with a similar structure increased stability and binding efficiency for RNA and proteins. Tetra-looped DsiRNAs were shown to have high biological activity in Dicer-expressing cells, while activity was reduced in Dicer-knockout cells. A number of studies have shown that short hairpin RNAs (shRNAs) provide a long-term effect of RNAi [64,65,66] due to the interaction of the duplex loop with Dicer’s N-terminal helicase domain [67,68,69,70] and its cofactor TRBP, which increases Dicer’s RNA binding affinity and enhances cleavage fidelity [71]. This suggests that the presence of a loop in small RNAs, as in tetra-looped DsiRNAs, may affect not only the Dicer cleavage activity but also the efficiency of target gene silencing. A comparison of the effectiveness of DsiRNA and looped DsiRNA showed that the presence of a hairpin in tetra-looped DsiRNA increases its biological activity from 4% to 18% when delivered to cells using a transfection agent (Table 3, #12).

One of the most promising approaches to date is the use of a fully modified tetra-looped DsiRNA conjugated with GalNac [72], which suppresses the expression of the LDHA gene by 98% in vitro and 50% in vivo (Table 3, #13).

Thus, DsiRNA bioconjugates have high biological activity in vitro and in vivo and can be more effective than canonical siRNA. Since DsiRNA processing significantly improves the biological activity of DsiRNA, it is important to take into account its specificity for non-canonical siRNAs with other structures.

**Table 3:** Structural modifications of DsiRNA.

#	Structure of siRNA—Sense Strand 5’-3’ (S) Above, Antisense Strand 3’-5’ (AS) Below. Pattern of Chemical Modifications ^1,2,3^	In Vitro				In Vivo			Ref.
		Cells/ Target	Delivery ^4^	Efficiency ^5^ ↑-Increase ↓-Decline		Model/ Target	Dose, Mode of Adm ^6^	Efficiency ↑-Increase ↓-Decline	
7	siRNA, n/m	HEK 293/*EGFP*	Lf2000 20 nM	~47%, IC50 ~30 nM, protein		-	-	-	[57]
	DsiRNA (25/27), n/m			~97%(↑50%), IC50 175 pM (↑×170), protein		-	-	-	
8	Palmitic acid (C16)-siRNA, n/m 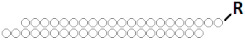 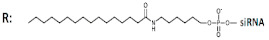	Hela/Rluc	Lf2000 1 nM	87%, Rluc exp		-	-	*-*	[58]
	Palmitic acid (C16)-siRNA (21/23), n/m 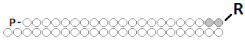 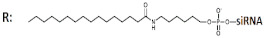			93% (↑6%), Rluc exp		-	-	*-*	
	Palmitic acid (C16)-DsiRNA (23/25), n/m 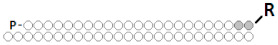 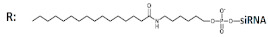			91% (↑4%), Rluc exp		-	-	*-*	
	Palmitic acid (C16)-DsiRNA (25/27), n/m 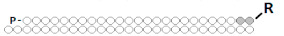 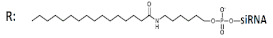			92% (↑5%), Rluc exp		-	-	*-*	
9	DsiRNA (25/27) 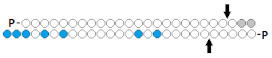	HEK293/ *CCR2*	RNAiMAX 1 nM	80%, mRNA		Rat, ganglion/*CCR2*	5 mcg, 2× intrathecal/Transductin	71%, mRNA	[59]
10	«RNA aptamer (A-1) to gp120»- DsiRNA 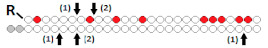	-	-	-	-	HIV-1 in mice^Rag2−/−γc−/−^	0.38 mg/kg, 8× i.v.	90%, mRNA (PBMC), 10^5^ fold (in plasma)	[60]
1	α- tocopherol -DsiRNA 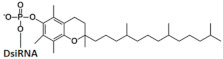 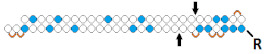	-	-	-	-	Mice, liver/*ApoB*	32 mg/kg, i.v.	80%, IC50 2 mg/kg, mRNA	[61]
13	DsiRNA 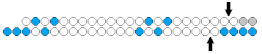	HeLa/ *LDHA*	RNAiMax 1 nM	~96.9%, mRNA		-	-	-	[72]
	Tetra-looped DsiRNA 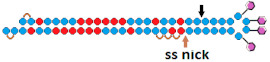 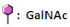			~98.5% (↑1.6%), IC50 20 пM, mRNA		Mice, liver/ *LDHA*	10 mg/kg, i.v.	50%, mRNA	
14	siRNA, n/m	Hela/Rluc	Lf2000 50 nM	48%, Rluc exp		-	-	-	[62]
	DsiRNA (27/27, “blunt” ends), n/m			83% (↑35%)		-	-	-	
	Cholesterol-DsiRNA (27/27, “blunt” ends), n/m 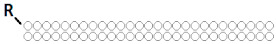 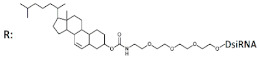		- 600 nM	30%, Rluc exp		-	-	-	
15	Palmitic acid (C16)-siRNA, n/m 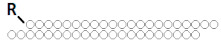 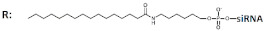	GCIY- eGFP/ *EGFP*	RNAiMAX 100 nM	75%, mRNA		Xenograft GCIY- eGFP in nude mice/ *EGFP*	3 nmol, i.t./Invivofectamine 2.0	62%	[63]
	Palmitic acid (C16)-DsiRNA (27/27, “blunt” ends), n/m 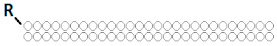 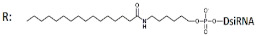			77%(↑2%), mRNA				70%(↑8%)	

^1^ Chemical modifications are indicated: 2’F (red), 2’O-Me (blue), 2’O-MOE (dark blue), dNMP (grey), PS (orange), 5’-(E)-VP (green), inverted thymidine (black), boranophosphate modification (purple) and LNA (yellow). n/m—non-modified. ^2^ “R”—linker with ligand. ^3^ Putative Dicer cleavage sites are indicated by black arrows. ^4^ “-”—delivery without transfection agent, Lf2000—transfection agent Lipofectamine 2000. ^5^ A decrease (↓) or increase (↑) in the effectiveness of a particular modification to silencing activity of siRNA. ^6^ Route of administration: i.v.—intravenously, s.c.—subcutaneously, i.t.—intratumoral injection, i.c.v.—intracerebroventricular.

## 8. Multimeric siRNAs

One of the unsolved problems in the development of siRNA-based drugs for clinical use is the rapid elimination of these molecules from the bloodstream due to filtration by the kidneys. Currently, various approaches are in development to increase the circulation time of siRNA in the bloodstream and their accumulation in target organs; however, these approaches have not yet been introduced into clinical practice and are limited to experimental studies [73,74,75]. It is assumed that the use of siRNA duplexes with a higher molecular weight as inducers of RNAi will improve the pharmacodynamic and pharmacokinetic properties of such drugs. Depending on the design of the duplex, long siRNAs may have a linear or branched structure; in the next section, we will take a closer look at each type of duplex architecture (Table 4 and Table 5).

### 8.1. Linear Multimeric siRNA Structures

Changing the structure of siRNA by increasing the molecular weight of the duplex makes it possible to increase the biological activity and, accordingly, reduce the dose of siRNA necessary to achieve the desired effect. Various approaches to the design of high molecular weight siRNA are actively discussed in the literature. The fundamental difference between these approaches is the method of connecting RNA: through a nucleotide linker or directly during synthesis, or through a non-nucleotide linker by chemical crosslinking.

Both approaches were compared in the work [76], where conjugates of GalNac and dimeric siRNA directed to the TTR and FVII genes were synthesized and their interfering properties were studied. These dimeric siRNA conjugates were characterized by an order of magnitude higher IC50 compared to a mixture of conjugates of monomeric siRNA when delivered to cells without a transfection agent (Table 4, #16). However, their biological activity in vivo was comparable to that of a mixture of monomeric siRNA conjugates. Thus, their advantage in the duration of circulation in vivo compensates for the lack of biological activity in vitro. It is assumed that the way monomeric siRNAs are connected can affect the flexibility of the duplex and, thus, the efficiency of interaction with RNAi proteins and suppression of the target gene. A comparison of the effectiveness of dimeric siRNA conjugates with linkers of different nature showed that the most active is the conjugate of dimeric siRNA with a nucleotide-based linker, which suppressed the expression of each of the two target genes by 92% in vivo.

Fully modified, PS-containing asymmetric siRNAs connected through a tetraethylene glycol linker were used in another study [77]. A comparison of the accumulation of Cy3-tagged dimeric and monomeric siRNA in mouse brain showed that dimeric siRNA accumulates in tissue much more efficiently than canonical siRNA, supporting the idea that cooperative interactions between two partially PS-modified siRNAs provide increased tissue accumulation. High accumulation of dimeric siRNA in brain structures correlated with the efficiency of target gene suppression. It has been shown that dimeric siRNA suppresses *Htt* gene expression by 90%, while PS-free dimeric siRNA is inactive. Based on these data, it can be concluded that the introduction of PS modification into the composition of single-stranded overhangs of asymmetric siRNAs can significantly improve their interfering properties.

The biodistribution and silencing activity of fluorescently labeled dimeric siRNA connected by an oligoethylengrycol linker were studied in Cynomolgus monkeys. A month after a unilateral injection into the brain ventricle, a uniform distribution of dimeric siRNA was observed throughout the primate brain, with the highest accumulation in the cortex (9 μg/g), hippocampus (6 μg/g) and thalamus (6 μg/g) and the lowest accumulation in the caudate nucleus (2 µg/g) and nucleus basalis (1 µg/g) of the striatum [77]. Effective suppression of the HTT protein by 90% in the cortex, 80% in the hippocampus, 50–85% in the caudate nucleus, and 40–70% in the striatal nucleus basalis was achieved using dimeric siRNA (Table 4, #17). Thus, the dimeric siRNAs studied in this work can be considered promising agents for their use in clinical practice for the treatment of diseases of the central nervous system.

On the other hand, there are a number of studies in which dimeric siRNA was obtained by connecting monomers through a nucleotide linker. For example, in [78], a 66% decrease in the expression of the *BIRC5* gene and 83% of the *BCL2* gene was achieved when dimeric siRNA was delivered to cells using a transfection agent (Table 4, #20). The rate of *BIRC5* gene suppression by monomeric siRNA was 55%, whereas suppression of the *BCL2* gene by dimeric siRNA was only slightly less effective.

The hybridization of long sense and antisense strands, which contain sequences of monomeric siRNAs directed to different targets, is a linker-free method for obtaining dimeric siRNAs. For example, in [79], dimeric siRNAs were designed to suppress two target genes, *LMNA* and *TIG3*; the silencing activity of the dimer for each target did not differ significantly from the activity of individual monomeric siRNAs and reached more than 90% when delivered to HeLa cells using a transfection agent (Table 4, #21).

A further increase in molecular weight for trimeric siRNAs was made in a study [80] comparing the interfering activity of a selectively modified 21 bp siRNA with that of 42 bp siRNA (dimeric siRNA) and 63 bp siRNA (trimeric siRNA), which contain two and three copies of the canonical siRNA sequence, respectively. It was shown that 6 days after transfection of KB-8-5 cells, trimeric siRNA suppresses *MDR1* gene expression more effectively than canonical and dimeric siRNA. The IC50 value of trimeric siRNA was two and 4.6 times lower compared to dimeric and canonical siRNA, respectively (Table 4, #22). The study of the mechanism of action of selectively modified dimeric and trimeric siRNAs showed that these duplexes are not subjected to Dicer cleavage. Despite the fact that the potential cleavage sites in the siRNAs studied in this work did not contain modifications, inhibition of Dicer cleavage may be associated with the presence of 2’O-methyl modifications in one or two nucleotides from the putative Dicer cleavage sites, which may prevent processing. An increase in the effectiveness of the action of extended siRNA variants in this case may be due to the fact that such siRNAs can directly be loaded in a complex with Ago2 and, independently of Dicer, participate in RNAi.

Since Dicer’s processing of extended siRNA variants can increase the concentration of functional siRNAs, it makes sense to optimize the pattern of chemical modifications so that the modifications do not interfere with this process. In addition, Dicer treatment may allow the use of extended siRNA variants to effectively suppress the expression of several genes at once. Thus in [81], a 63-bp trimeric siRNA was investigated, containing in one duplex sequences directed to the mRNA of the *MDR1*, *LMP2*, and *LMP7* genes with a limited number of 2’O-Me modifications at nuclease-sensitive sites. It has been shown that this siRNA is able to suppress the expression of all three target genes with high efficiency, acting through a Dicer-dependent mechanism. In the cell, this trimeric siRNA is cut into duplexes 42 and 21 bp long and suppresses the expression of target genes much more effectively than canonical siRNA with the corresponding monomer sequence.

A study of the properties of cholesterol derivatives of 63 bp trimeric siRNAs consisting of three copies of the canonical siRNA [8] showed that an increase in the length of the siRNA duplex in the cholesterol conjugate increases its biological activity when delivered to KB-8-5 cells using a transfection agent. However, upon delivery in a carrier-free mode, the efficiency of accumulation of conjugates in cells and the efficiency of suppression of the target gene are significantly reduced. An in vitro study of the biological activity of the conjugates showed that the canonical siRNA conjugate suppresses *MDR1* gene expression by 50%, while the trimeric siRNA conjugate is inactive (Table 4, #23). In vivo experiments on a mouse model with a subcutaneously grafted KB-8-5 tumor showed that the cholesterol conjugate of monomeric siRNA effectively suppresses the expression of the target gene, while the cholesterol conjugate of trimeric siRNA has no biological activity, despite efficient accumulation in xenograft tumor cells. The lack of correlation between the level of accumulation and biological activity can be explained by the inefficient release of the cholesterol conjugate of trimeric siRNA from endosomes into the cell cytoplasm, where RNAi occurs.

**Table 4:** Structural modifications of linear multimeric siRNA.

#	Structure of siRNA—Sense Strand 5’-3’ (S) Above, Antisense Strand 3’-5’ (AS) Below. Pattern of Chemical Modifications ^1,2,3^	In Vitro			In Vivo			Ref.
		Cells/ Target	Delivery ^4^	Efficiency ^5^ ↑-Increase ↓-Decline	Model/ Target	Dose, mode of Adm ^6^	Cells/ Target	
16	siRNA mix 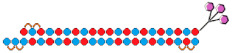 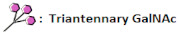	Prim. hepatocytes/*TTR/FVII*	-	IC50 0.1 nM (*TTR*) IC50 ~0.25 nM (*FVII)*	Mice liver/ *TTR*/ *FVII*	3 mg/kg, s.c.	~98%/~98%	[76]
	Dimeric siRNA 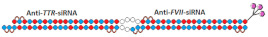			IC50 0.4 nM (↓×4)/~1 nM(↓×4)		3 mg/kg, s.c.	92%(↓6%)/92% (↓6%)	
	Dimeric siRNA 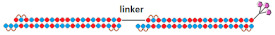 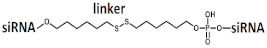			IC50 1 nM (↓×10)/IC50 1 nM (↓×4)		3 mg/kg, s.c.	73%(↓25%)/~82%(↓16%)	
17	siRNA 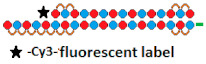	HeLa/*HTT*	RNAiMax 0.1 nM	70%, IC50 ~25 pM, mRNA	-	-	-	[77]
	Dimeric siRNA 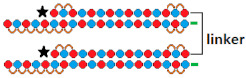 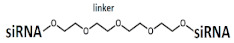			80%, (↑10%) IC50 ~10 pM (↑×2.5), mRNA	Mice, brain/ *HTT*	23.75 mg/kg, i.c.v. bilaterally	>90%, protein in the hippocampus	
							>70%, protein, in the cortex	
					M.Cynomolgus brain	0.017 mg/kg, i.c.v.	>90%, protein, in the cortex	
18	GalNAc-siRNA 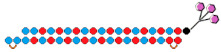	-	-	*-*	Mice, liver/*FVII*	50 mg/kg, s.c.	85%, protein	[82]
	GalNAc-dimeric siRNA 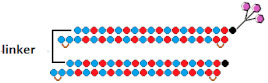 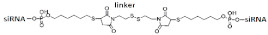	-	-	*-*			75%(↓10%), protein	
19	GalNAc-siRNA mix	-	-	*-*	Mice, liver/*FVII/ApoB/TTR*	50 mg/kg, s.c.	50%/30%/97%	
	GalNAc-trimeric siRNA 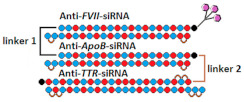 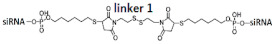 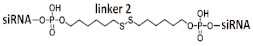	-	-	*-*			62% (↑12%)/30%/97%	
20	siRNA 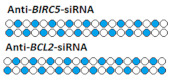	T24/*BIRC5/BCL2*	Lf2000 10 nM	55%/85%,mRNA	-	-	*-*	[78]
	Dimeric siRNA 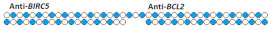			66% (↑11%)/83% (↓2%), mRNA	-	-	*-*	
21	siRNA mix	Hela/*LMNA/* *TIG3*	Lf2000 10 nM	90%/98%, mRNA	-	-	*-*	[79]
	Dimeric siRNA (34 bp), n/m 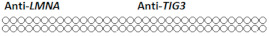			98% (↑8%)/96% (↓2%), mRNA	-	-	*-*	
22	siRNA 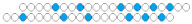	KB-8-5/*MDR1*	Oligofectamine	IC50 23 nM	-	-	*-*	[80]
	Dimeric siRNA 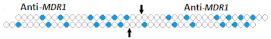			IC50 10 nM (↑×2.3)	-	-	*-*	
	Trimeric siRNA 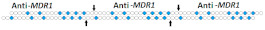			*MDR1*, IC50 5 nM (↑×4.6)	-	-	*-*	
23	Cholesterol-siRNA 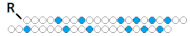 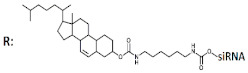	KB-8-5-MDR1-GFP/*MDR1*	Lf2000	IC50 29 nM	Xenograft KB-8-5 in SCID mice/*MDR1*	8.5 mg/kg i.v.	60%, protein	[8]
			- 5 mcM	48%, protein				
	Cholesterol—trimeric siRNA 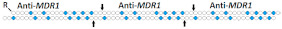 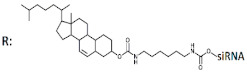		Lf2000	IC50 16 nM (↑×1.8)			0% (↓60%), protein	
			- 5 mcM	2%(↓46%), protein				

^1^ Chemical modifications are indicated: 2’F (red), 2’O-Me (blue), PS (orange), 5’-(E)-VP (green), inverted thymidine (black). n/m—non-modified. ^2^ “R”—linker with ligand. ^3^ Putative Dicer cleavage sites are indicated by black arrows. ^4^ “-”—delivery without transfection agent, Lf2000—transfection agent Lipofectamine 2000. ^5^ A decrease (↓) or increase (↑) in the effectiveness of a particular modification to silencing activity of siRNA. ^6^ Route of administration: i.v.—intravenously, s.c.—subcutaneously, i.t.—intratumoral injection, i.c.v.—intracerebroventricular.

The results of these studies show that an increase in the molecular weight of the duplex and construction of trimeric siRNAs make it possible to achieve a high level of accumulation in the target organ; however, for biological activity in the suppression of the target gene, long siRNAs must leave endosomes. The efficiency of the escape of long siRNAs from endosomes can be affected by the design of such extended siRNA variants. For example, trimeric siRNAs were constructed in [82] by connecting monomers through a cleavable linker. In vivo experiments have shown that the conjugate of GalNac and trimeric siRNA containing sequences directed to the *FVII*, *ApoB*, and *TTR* genes actively suppresses the expression of these target genes by 62%, 30%, and 97%, respectively. The biological activity of the mixture of monomeric siRNA conjugates was similar in suppression of the *ApoB* and *TTR* genes and 12% lower in suppression of the *FVII* gene (Table 4, #19). It can be assumed that the use of a cleavable linker both increases the flexibility of the long siRNA structure and leads to the release of monomeric siRNAs after linker cleavage and, thus, promotes better exit from endosomes into the cell cytoplasm.

Thus, changing the structure of siRNA by increasing the molecular weight and introducing chemical modifications provides highly specific and long-term inhibition of target gene expression without causing a toxic effect. However, a balance between high molecular weight and efficiency of escape from endosomes is required to achieve a high silencing effect.

### 8.2. Branched Multimeric siRNA Structures

Another multimeric siRNA variant with increased molecular weight is branched siRNA, which contains several copies of siRNAs directed to the same or different target genes. One of the proposed branched siRNAs deployed a structure consisting of three siRNAs interconnected by a linker based on deoxyribonucleotides [83]. This branched siRNA was shown to be 4.7 times more efficient than canonical siRNA when delivered to Hela cells with a transfection agent (Table 5, #24). It is assumed that such branched siRNAs can be processed by Dicer, which leads to an increase in their efficiency.

**Table 5:** Structural modifications of branched multimeric siRNA.

#	Structure of siRNA—Sense Strand 5’-3’ (S) Above, Antisense Strand 3’-5’ (AS) Below, Pattern of Chemical Modifications ^1,2,3^	In Vitro			In Vivo			Ref.
		Cells/ Target	Delivery ^4^	Efficiency ^5^ ↑-Increase ↓-Decline	Model/ Target	Dose, Mode of Adm ^6^	Cells/ Target	
24	siRNA, n/m	Hela/ *BIRC5*	Lf2000	IC50 3.89 nM, mRNA	-	-	*-*	[83]
	Branched siRNA (36 n.), n/m 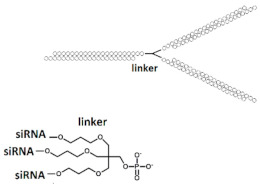			IC50 0.83 nM (↑×4.7), mRNA	-	-	*-*	
25	siRNA mix	HeLa/*LMNA/* *DBP/TIG3*	Lf2000	60%/47%/82%, Rluc exp	-	-	-	[84]
	Branched siRNA (38 n.) 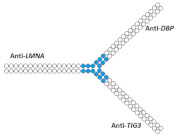			68% (↑8%)/62% (↑15%)/85% (↑3%)	-	-	-	
26	siRNA mix	Hela/*BIRC5/CTNNB/MET*	Lf2000	76%/77%/90%, Rluc exp	-	-	-	[85]
	Branched siRNA (38 n.), n/m 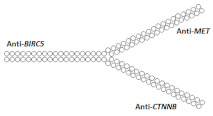			85% (↑9%)/45% (↑32%)/77% (↓13%)%, Rluc exp	-	-	-	
27	siRNA, non-modified	Hepa 1–6/*ApoB*	PEI-Gal 100 nM	50%, mRNA	Mice, liver/*ApoB*	6 mg/kg, i.v. with PEI-Gal	33%, mRNA	[86]
	Branched siRNA (32 n.), n/m 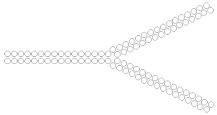			*ApoB* 70% (↑20%), mRNA			63% (↑30%), mRNA	
28	siRNA, n/m	Hela/Rluc	RNAiMax	78%, Rluc exp	-	-	*-*	[87]
	Branched siRNA (21 n.), n/m 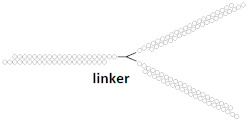 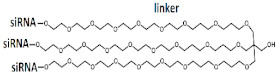			81% (↑3%), Rluc exp	-	-	*-*	
29	siRNA mix	Hela/*BIRC5/CTNNB/* *STAT3/* *MET*	PEI 25 nM	39%/30%/40%/30%, mRNA, 1 d.	-	-	*-*	[88]
	Branched siRNA (38 n.) 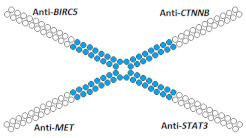			61% (↑22%)/40% (↑10%)/70% (↑30%)/62% (↑32%), mRNA	-	-	*-*	
30	siRNA, n/m	SMMC-7721/ *BIRC5/BCL2*	Lf2000	80%/70%, protein, 2 d.	-	-	*-*	[89]
	Dimeric siRNA 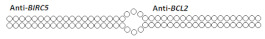			48% (↓32%)/45% (↓25%), protein	-	-	*-*	
	Dimeric siRNA 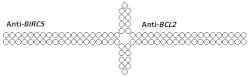			46% (↓34%)/57% (↓13%), protein	-	-	*-*	
	Trimeric siRNA 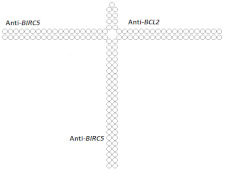			68% (↓12%)/48% (↓22%), protein	-	-	*-*	

^1^ Chemical modifications are indicated: 2’O-Me (blue), n/m—non-modified. ^2^ “R”—linker with ligand. ^3^ Putative Dicer cleavage sites are indicated by black arrows. ^4^ “-”—delivery without transfection agent, Lf2000—transfection agent Lipofectamine 2000. ^5^ A decrease (↓) or increase (↑) in the effectiveness of a particular modification to silencing activity of siRNA. ^6^ Route of administration: i.v.—intravenously.

On the other hand, siRNA copies can be linked directly via a phosphodiester bond. It was shown in the following studies [84,85,86] that siRNA with such a branched structure exhibits high efficiency compared to the canonical siRNA in in vitro experiments (Table 5, #25–27). The mechanism of action of such branched siRNAs revealed that Dicer-mediated processing is not required for gene silencing and that RNAi is triggered by a 38 nucleotide long guide strand [84]. In order to evaluate the therapeutic potential of branched siRNAs in vivo, mice were intravenously injected with 6 mg/kg of branched siRNA complexed with a conjugate of polyethyleneimine and galactose (PEI-Gal) [86]. It was shown that 2 days after the administration of the complexes, the mRNA level of the *ApoB* gene decreased by 63% and 33% respectively, in the case of branched and canonical siRNA (Table 5, #27).

Another option for the design of branched siRNAs is the connection of monomeric siRNAs through a hexaethylene linker [87]. These molecules are stable in serum and have no off-target effects. The study of biological activity showed that the effectiveness of such branched siRNAs in vitro was 81% and was comparable to the efficiency of canonical siRNAs (Table 5, #28). Taken together, the results of this study suggest that such branched siRNAs are promising for in vivo use.

During the study of the interfering properties of branched siRNA, consisting of the monomers directed to four different genes [88], it was shown that the efficiency of such branched siRNA in suppressing the target genes *BIRC5*, *CTNNB*, *STAT3*, and *MET* is 20–30% higher than canonical siRNA mixtures (Table 5, #29). Thus, it is possible to further increase the molecular weight and to design siRNA with a more complex structure.

To increase the stability of long siRNAs in [89], it was proposed to construct siRNA with a secondary structure of a “loop” or “clover leaf”. It has been shown that such siRNAs are able to suppress the expression of several target genes at once, acting along the Ago2-dependent pathway without causing undesirable immunostimulation. However, the efficiency of siRNAs with such secondary structures was lower than the efficiency of a mixture of canonical siRNAs when delivered to cells with a transfection agent (Table 5, #30). The data obtained in this work suggest that siRNAs with various structural modifications can induce RNAi. However, the question of whether such siRNA constructs solve the problem of rapid removal of siRNA from the body remains open.

Thus, an increase in the molecular weight of siRNA partially solves the problem of their rapid excretion from the body by filtration by the kidneys and contributes to a high accumulation of siRNA in the target organ, but does not always guarantee a high level of activity in suppressing target gene expression since it significantly hinders the release of siRNA from endosomes into the cytoplasm. Therefore, changing the structure and molecular weight of siRNA in order to find a balance between the effective accumulation of siRNA in the cytoplasm of the target cell and its slow excretion from the body remains an urgent task.

## 9. Conclusions

The described approaches for increasing the molecular weight of siRNA make it possible to increase the circulation time of siRNA in the blood and, consequently, biodistribution. However, for the effective realization of multimeric siRNA, it must be combined with systems that improve the release of siRNA from endosomes. Since no non-toxic in vivo agent has yet been found to escape endosomes, the most promising approach is the use of multimeric siRNAs with cleavable bonds between themselves. Such dsRNAs will be cleaved to monomeric siRNAs, which do not have the same poor endosomal escape as multimeric siRNA. Another important issue for increasing multimeric siRNA efficiency is optimization of the 2′F/2′O-Me composition in fully modified multimeric siRNA. It should be noted that for fully modified dsRNAs, processing to monomeric siRNAs can be blocked in the presence of 2’O-Me at Dicer’s cleavage sites, and Dicer-independent loading might not be very efficient in this case; so, it is necessary to use deoxyribonucleotide or 2’F modifications at Dicer’s cleavage sites. Unexpectedly, fully modified DsiRNA from Dicerna Pharma does not require Dicer’s cleavage activity because it contains a non-cleavable 2’O-Me modified nucleotide at the site of Dicer’s cleavage and still has high efficiency silencing activity. Processing of such DsiRNA occurs in a Dicer-independent manner, and Dicer can solely direct the loading of siRNA into RISC [71]. Another benefit from the DsiRNA structure is the additional protection from nucleases provided by the tetraloop hairpin, which is the most important factor for the long-term silencing activity of siRNA. Thus, combining structural siRNA modification with rational chemical modification design will improve biodistribution and reduce therapeutic dose, allowing the development of new treatments for previously incurable diseases.

## 10. Future Prospects

Future prospects for the extrahepatic use of siRNA are successful using Alnylam’s advanced chemical modification patterns IKARIA and their lipophilic monomeric siRNAs, which may be in clinical practice in a few years for treating diseases of the lungs, brain, and muscles or other organs [74,75,90,91]. The most effective structural modification for extrahepatic use is a dimeric siRNA from Atalanta Therapeutics [77], which after a single injection silences the target gene in the brain for several months; the company is developing this platform, so in the future we can expect the release of numerous drugs with improved chemical patterns of modifications and aimed at various diseases of the brain. Thus, the use of siRNA in the clinic is progressing toward reducing the cost of treatment due to new chemical modifications and increasing the number of new drugs and, accordingly, the impact that this technology has on the standard of living of humankind.

## Data Availability

Publicly available datasets were analyzed in this study.
